# Application of 18F-FDG PET/CT imaging in a primary angiomatoid fibrous histiocytoma of pulmonary bronchus: case report and literature review

**DOI:** 10.3389/fmed.2024.1415042

**Published:** 2024-07-31

**Authors:** Mingyan Shao, Sisi Fan, Wanling Qi, Zhehuang Luo, Rong Xu, Fengxiang Liao

**Affiliations:** ^1^Department of Nuclear Medicine, Jiangxi Provincial People’s Hospital, The First Affiliated Hospital of Nanchang Medical College, Nanchang, Jiangxi, China; ^2^Department of Pathology, Jiangxi Provincial People’s Hospital, The First Affiliated Hospital of Nanchang Medical College, Nanchang, Jiangxi, China

**Keywords:** 18F-FDG PET/CT, CT, angiomatoid fibrous histiocytoma, pulmonary bronchus, primary

## Abstract

**Background:**

Angiomatoid fibrous histiocytoma (AFH) is a clinically rare, low-grade malignant soft tissue tumor that occasionally metastasizes. It accounts for 0.3% of all soft tissue tumors and most frequently occurs in the extremities, followed by the trunk, and the head and neck. Primary angiomatoid fibrous histiocytoma (PAFH) of the pulmonary bronchus is rare. In this paper, the clinical and imaging data of a case of PAFH of the pulmonary bronchus are reported, and the literature is reviewed.

**Case description:**

A 57-year-old female patient presented with a six-month history of cough without apparent cause, characterized by paroxysmal dry cough, chest tightness, and shortness of breath, which worsened with activity. She did not experience fever, chills, chest pain, hemoptysis, or night sweats. Laboratory tests revealed an elevated C-reactive protein and ferritin levels, while tumor markers such as AFP, CEA, CA199, CA125, CA50, and T-SPOT were negative. A chest CT scan showed bronchial obstruction, atelectasis, and a soft tissue density in the right middle lobe of the lung. The enhanced scan demonstrated uneven enhancement of endobronchial nodules. An 18F-FDG PET/CT scan revealed a nodular soft tissue density shadow in the right lung bronchus with uneven density, clear boundaries, and increased 18F-FDG uptake, with a maximum standard uptake value (SUVmax) of 11.2. Bronchoscopy revealed a nodular or polypoid mass that was yellow and tough. Based on imaging findings, the preoperative diagnosis favored lung cancer. However, the postoperative pathological diagnosis confirmed primary angiomatoid fibrous histiocytoma (PAFH) of the pulmonary bronchus.

**Conclusion:**

The incidence of primary angiomatoid fibrous histiocytoma (PAFH) is very low, and its clinical manifestations and imaging findings lack specificity, with the final diagnosis relying on pathology. PET/CT imaging has a certain value in the diagnosis of PAFH and holds significant application value in preoperative staging, postoperative efficacy evaluation, and follow-up monitoring. In conclusion, this case report further expands the spectrum of lung and bronchial tumors.

## Introduction

Angiomatoid fibrous histiocytoma (AFH) was originally named angiomatoid malignant fibrous histiocytoma as a subtype of fibrous histiocytoma and was first described by Enzinger in 1979 (1). However, subsequent reports have indicated that AFH behaves as a low-grade malignant or borderline lesion. The 1994 edition of the WHO Soft Tissue Classification categorized it as an intermediate fibrous histiocytoma. Later, due to the unclear differentiation direction of tumor cells, the 2002 edition of the WHO Soft Tissue Classification grouped it into tumors with uncertain differentiation (2). AFH is a rare low-grade malignant soft tissue tumor that occasionally metastasizes, comprising 0.3% of all soft tissue tumors. It most commonly occurs in the extremities, followed by the trunk, and the head and neck (3). Pulmonary angiomatoid fibrous histiocytoma (PAFH) is rare, and its pathogenesis is not well understood. It can occur in the pulmonary lobes, pulmonary arteries, and bronchus (4, 5). Its clinical manifestations and imaging findings lack specificity, and the final diagnosis relies on pathology. PAFH is relatively rare, with few clinical reports and even fewer accounts of PET/CT imaging findings. This paper presents the PET/CT imaging features of a case of PAFH. The clinical, imaging, and pathological data of the case are summarized, and the relevant literature is reviewed to enhance the understanding and diagnostic capability of this disease and to provide assistance for future patient management.

## Case description

**Figure 1:**
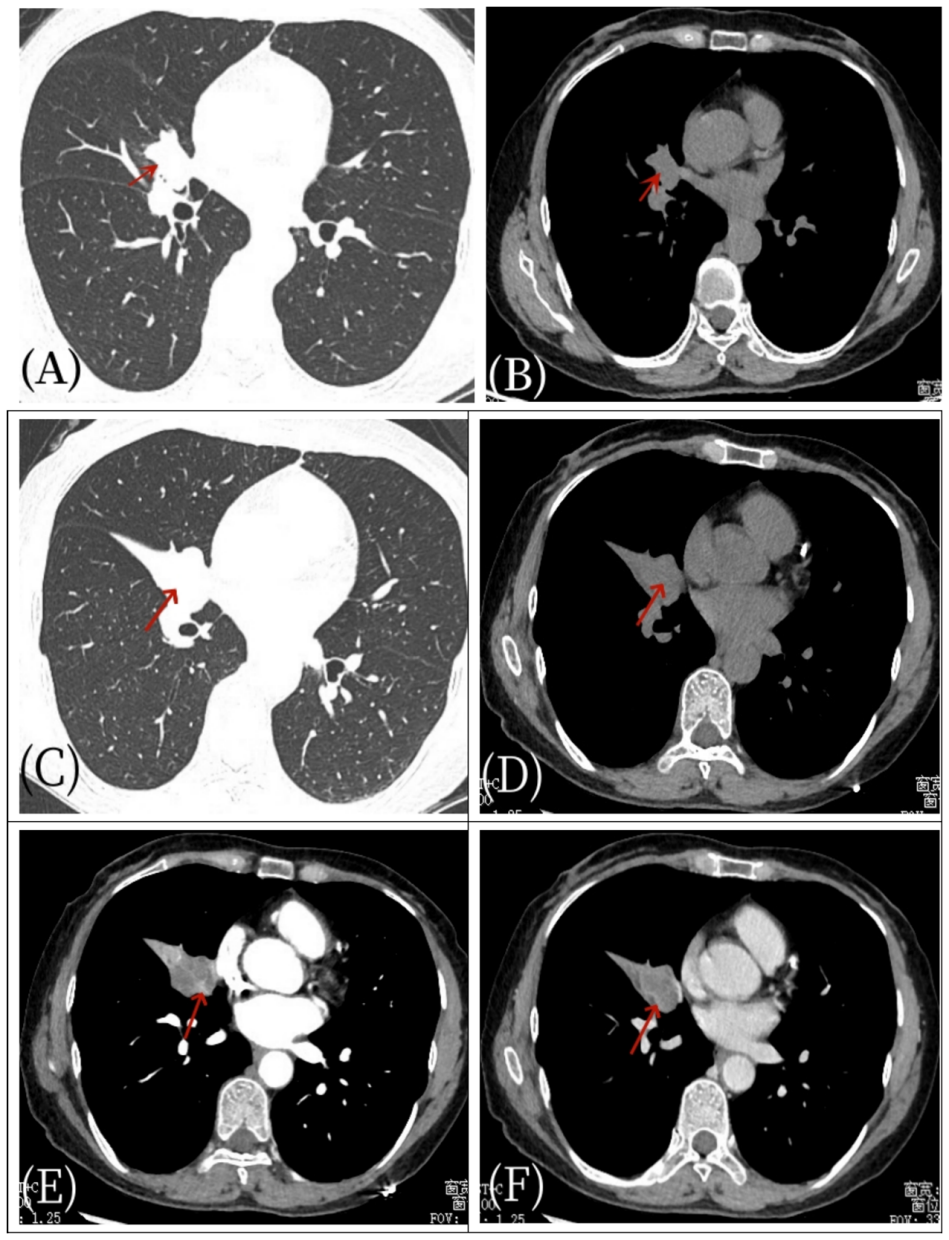
Female, 57 years old, pulmonary angiomatoid fibrous histiocytoma. On April 27, 2021, a non-contrast CT **(A,B)** scan showed a space within the bronchus of the right middle lobe (arrow) without evidence of obstructive atelectasis or pneumonia. After 1 week, the patient’s condition worsened, manifesting as pulmonary atelectasis. Subsequent axial non-contrast CT scan **(C,D)** and axial CT enhanced scan **(E,F)** revealed right middle lobe bronchial obstruction, atelectasis with a soft tissue density shadow (arrow), and significant enhancement of the mass (arrow) as well as mediastinal enlarged lymph node shadows.

**Figure 2:**
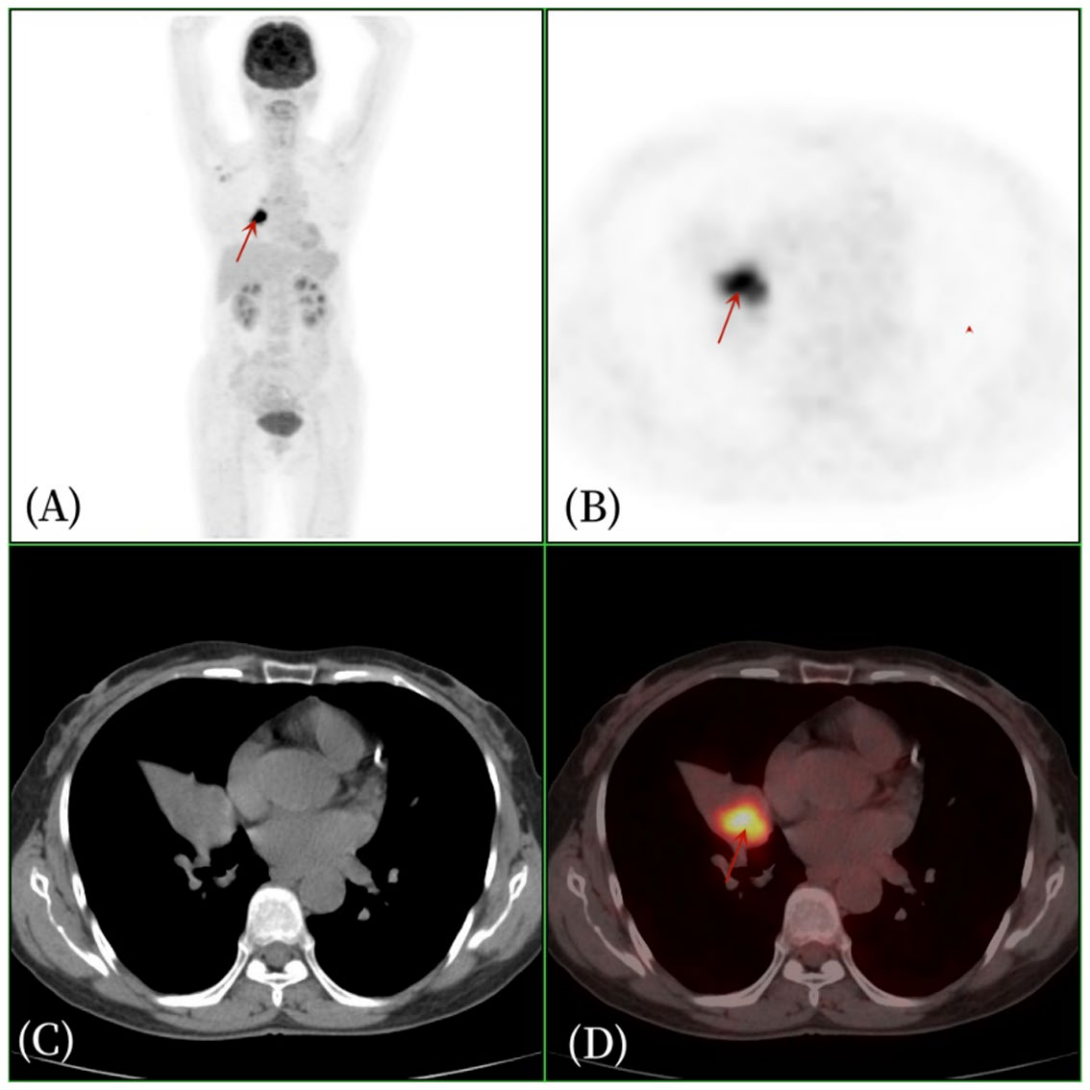
Female, 57 years old, pulmonary angiomatoid fibrous histiocytoma. **(A)** whole body MIP, **(B)** axial PET, **(C)** axial CT, **(D)** axial fusion. The 18F-FDG PET/CT revealed right middle lobe atelectasis and a 2.2 cm × 2.5 cm soft tissue density nodule within the bronchus of the right middle lobe of the lung. The density was uneven, with clear boundaries, and there was increased glucose metabolism, with an SUVmax of 11.2 (arrow).

**Figure 3:**
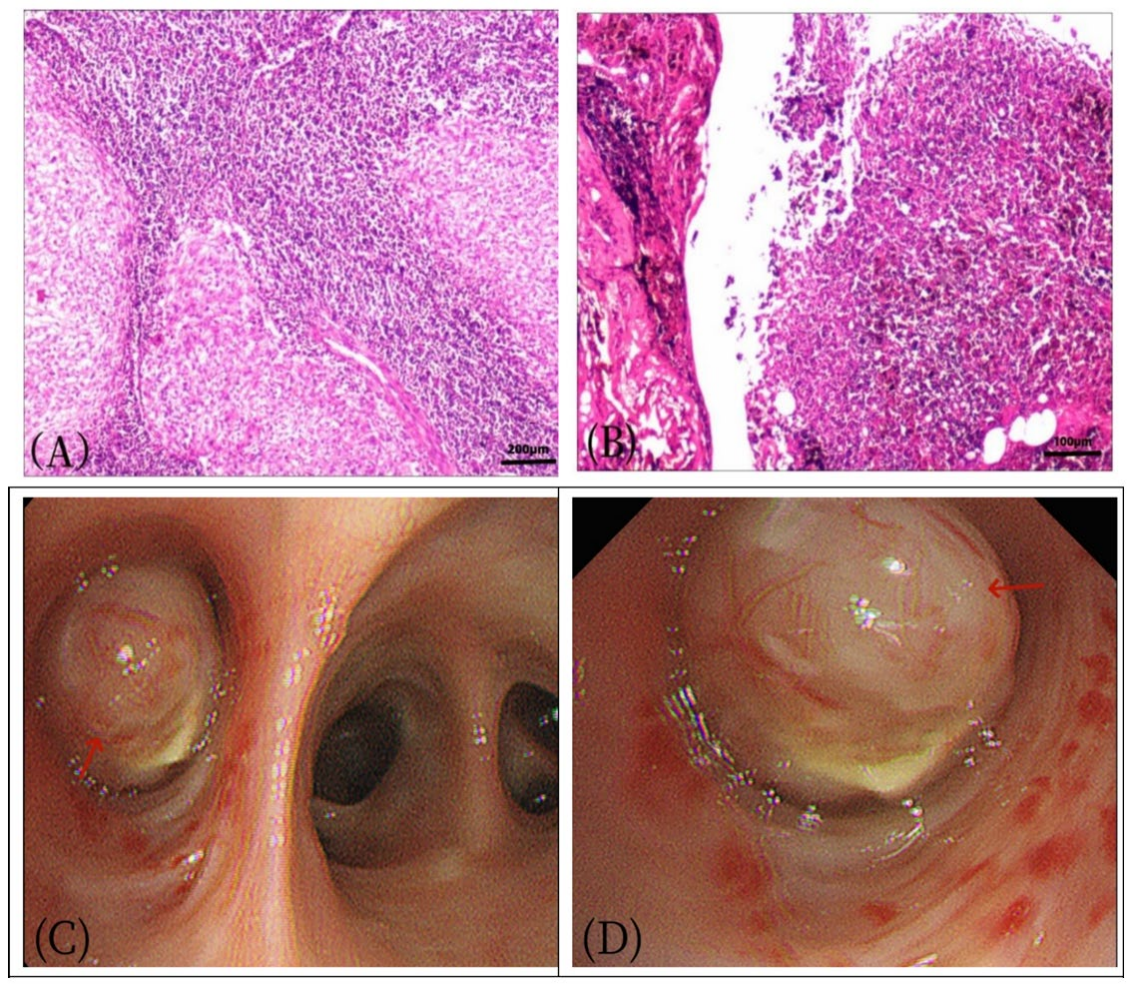
Female, 57 years old, pulmonary angiomatoid fibrous histiocytoma. **(A)** HE×100, **(B)** HE×200. The tumor exhibited mild cell morphology, with fusiform and oval nuclei. Microscopically, the tumor appeared nodular, containing irregular pseudovascular spaces, and some tumor cells displayed atypia. **(C,D)** A nodular mass (arrow), measuring approximately 2.0 × 3.0 cm, was observed in the bronchus adjacent to the bronchial incision, characterized by its yellow and tough texture.

Three years ago, a 57-year-old woman presented to Jiangxi Provincial People’s Hospital with symptoms of dry cough, chest tightness, and shortness of breath, which worsened with activity. She reported no fever, chills, chest pain, hemoptysis, or night sweats. She denied any personal history of tumor and a family history of genetic medical conditions. The patient did not initially seek further medical attention and chose to treat herself with Chinese medicine at home, which provided some relief for the dry cough, but the symptoms persisted and recurred. The patient underwent a CT examination in the outpatient department of our hospital before starting any medical treatment (Figure 1). As depicted in Figure 1, a nodular mass was identified in the middle lobe of the right lung. After 1 week, the patient’s cough recurred and intensified without any apparent trigger, presenting with a dry cough, and there was no hemoptysis, fever, or chills. Laboratory tests: White blood cell count 7.6 × 109/L↑, eosinophilic percentage 8.6%↑, C-reactive protein 50.9 mg/L↑, ferritin 346 ng/mL↑, AFP (−), CEA (−), CA199 (−), CA125 (−), CA50 (−), T-SPOT (−). Imaging examination: The chest CT scan (Figure 2) revealed a nodular soft tissue density shadow in the bronchus of the right middle lobe, accompanied by bronchial obstruction and atelectasis of the right middle lobe. The enhanced scan showed mild to moderate uneven enhancement, with slightly enlarged lymph nodes in the mediastinum. The 18F-FDG PET/CT imaging (Figure 3) demonstrated a nodular soft tissue density shadow in the bronchus of the right middle lobe of the lung, with uneven density, clear boundaries, and a size of approximately 2.2 cm × 2.5 cm. The 18F-FDG uptake was more uniform, and the maximum standard uptake value (SUVmax) was 11.2. There were several slightly enlarged lymph nodes in the right hilum and mediastinum, with the largest group having a diameter of about 1.0 cm, showing slightly increased 18F-FDG uptake, and an SUVmax of 2.8. The PET/CT was initially misdiagnosed as lung cancer, with the lymph nodes considered reactive hyperplasia. Bronchoscopy (Figure 3) revealed a nodular mass at the opening of the right middle lobe of the lung, blocking the lumen, and the possibility of bronchial lung cancer was considered. Microscopic biopsy results showed histocytes and dendritic cells in a background of lymphocytes and plasma cells, with no components of squamous cell carcinoma. Following a multidisciplinary consultation, the patient elected to undergo surgical treatment. The postoperative pathology (Figure 3) revealed an intermediate soft tissue tumor in the middle lobe of the right lung, considered to be pulmonary angiomatoid fibrohistiocytoma. Immunohistochemistry results were as follows: CK (−), vimentin (+), ALK (−), desmin (partial +), CD31 (+), S-100 (a little +), CD163 (a little +), CD34 (−), ERG (−), SMA (−), Ki-67 (+, about 15%). In follow-up, the patient, without adjuvant therapy, recovered well after surgery, with no significant cough or sputum and no other discomfort. A PET/CT review 1 year later showed no recurrence or metastasis, and subsequent annual CT scans also revealed no recurrence.

## Discussion

Pulmonary angiomatoid fibrous histiocytoma (PAFH) is rare, and its pathogenesis is not well understood, though it may be linked to genetic factors. AFH is characterized by the presence of fusion genes resulting from translocation mutations involving three related genes: EWSR1-CREB1, EWSR1-ATF1, and FUS-ATF1. Disruption of the EWSR1 gene is detected in the majority of cases, although other fusion genes may also be present (6, 7). Fluorescence *in situ* hybridization (FISH) detection was performed in 7 out of 9 cases of pulmonary AFH, and EWSR1 gene disruption was detected in all cases. As reported in the literature, molecular detection of Ewing sarcoma breakpoint region 1 (EWSR1) gene rearrangement is positive in more than 90% of cases (8), indicating its significant role in diagnosis. In the case of the patient presented here, genetic testing was not conducted due to family economic constraints.

Angiomatoid fibrous histiocytoma (AFH) primarily affects children and young adults, and is rare in individuals over 40 years old. It most frequently occurs in the limbs, followed by the trunk, head, and neck. AFH can also arise in the lung, mediastinum, retroperitoneum, vulva, ovary, bone, and other locations, accounting for only 0.3% of soft tissue tumors (8–10). Pulmonary angiomatoid fibrous histiocytoma (PAFH) is particularly rare. Clinically, it often presents as a painless mass and rarely metastasizes. Symptoms vary depending on the tumor’s location, with some patients exhibiting cough, sputum, chest tightness, hemoptysis, and dyspnea, while others may be asymptomatic. Imaging findings for PAFH are non-specific (Table 1), and the tumor is often characterized by localized, multinodular, or polycystic hemorrhagic masses. The cystic cavities formed by repeated bleeding can lead to misdiagnosis as hematomas or hemangiomas (11, 12). PAFH has certain pathological features, including histiocytoid cells and irregular pseudovascular spaces, surrounded by variable lymphocyte or plasma cell infiltration, occasional mesenchymal mucous degeneration, and often a dense fibrous pseudocapsule. The tumor cells are arranged in clusters, matting stripes, or whorls, with oval or fusiform nuclei, mild atypia, and rare mitotic figures or necrosis. PAFH lacks a characteristic immunophenotype, with vimentin being the only diffuse marker in all PAFH cases, while EMA, calponin, desmin, CD99, and CD68 may be expressed to varying degrees (7, 13). Approximately one-third of PAFH cases lack the classical hemangiomatoid structure and grow as nodules, referred to as the solid type (14). It has been reported (15) that no bleeding sac was found in the interstitium of PAFH in three such cases. Chen et al. (3) reported on PAFH occurring in the mediastinum, retroperitoneum, lung, ovary, and bone, with more common mucous degeneration. In this case, the myxoid degeneration between some tumor cells in PAFH is obvious, which is consistent with the pathological characteristics of PAFH.

**Table 1:** Literature reports of AFH of 18F-FDG PET / CT in foreign language in recent years.

Author	Age (years)	Gender	Sign of 18F-FDG PET/CT or CT	Metastasis	Treatment and Survival at the time of report
Luo et al. (11)	57	Female	PET/CT revealed a right parahilar nodule with intense FDG-avidity, middle lobe atelectasis, and several bilateral axillary lymph nodes with mild hypermetabolic activity.	NO	2 years post operation, follow-up indicated that this patient recovered well without evident recurrence of tumor
Haug et al. (12)	39	Male	PET/CT demonstrated marked hypermetabolism of the mass lesion.	NO	Without reporting
Çetin et al. (13)	29	Male	CT showed a mass in the lower lobe of the left lung	Without reporting	Received adjuvant chemotherapy
Tay et al. (14)	70	Female	CT scan showing a nodule in the upper lobe of the right lung.	Lymph node metastasis.	Without reporting
Kobayashi et al. (7)	54	Male	FDG-PET showed uptake at a bone tumor at the scapula with destruction of cortical bone and invasion into soft tissue	NO	There was no recurrence or metastasis 5 years after the treatment.
Makis et al. (15)	29	Female	Showed multiple foci of intense FDG uptake in the left shoulder and left axillary regions. The SUVmax is 6.2 and4.4	Lymphatic metastasis	The patient was given preoperative adjuvant chemotherapy (ifosfamide, mesna, etoposide). The mass and nodules were then surgically excised.
Colangeli et al. (16)	15	Female	PET/CT showed an uptake in the soft tissue of the leg (SUVmax 3.2) without bone involvement	NO	Wide surgical excision andthorough follow-ups are recommended in the management of this disease. No evidence of disease was present at the 18-month follow-up.
Gu et al. (17)	7	Male	PET-CT evaluation showing high FDG uptake in a left inguinal lymph node and significant FDG uptake in a left thigh lesion	Lymphatic metastasis	Surgical excision
Engkebølle et al. (18)	50	Male	With equivocal 18F-FDG PET/CT findings but with subsequent highly increased metabolic activity using 18F-FET PET/CT confirming tumor recurrence.	NO	Tumor recurrence.
Gui et al. (19)	49	Male	PET/CT scan shows an intensely fluorodeoxyglucose-avid right lower lobe mass. CT scan shows a mass in the right lower lobe of the lung. From the mass, there is an endobronchial extension component medially with occlusion of a subsegmental bronchus.	NO	A right lower lobe lobectomy was performed. The patients were followed up with no evidence of residual or recurrent tumor for 28 months.
Thway et al. (20)	64	Male	PET/CT showed a 15-mm lesion in the left lower lobe that was mildly fluorodeoxyglucose (18 F) avid	NO	The patient underwent resection of the left lower lobe, with uneventful recovery.
Thway et al. (20)	61	Male	PET/CT showed a polypoid tumor in the distal right main bronchus, that was intensely fluorodeoxyglucose (18 F) avid.	NO	The patient subsequently a sleeve resection of the lesion was performed, with uneventful recovery.
Gui et al. (19)	44	Male	CT chest confirmed a solid mass in the left main bronchus. Grossly, this was a solitary well circumscribed nodular mass. The tumor was centered around the bronchus and extensively involved the submucosa.	NO	A left upper lobectomy was performed. The patients were followed up with no evidence of residual or recurrent tumor for 5 years
Tay et al. (14)	70	Female	A staging CT scan of the chest found an indeterminate nodule in the right upper lobe of the lungs.	Mediastinal lymph node metastasis	She underwent video assisted thoracic surgery (VATS) with wedge resection of the lung nodule and excision of the lymph node.
Ghigna et al. (21)	76	Female	An enhanced CT scan showed a lesion at the origin of the right pulmonary artery, bulging into the vascular lumen, and extending to the emergence of the first mediastinal artery. This lesion was well delineated, solid, and moderately hypervascularized. PET/CT revealed significant 18 FDG uptake.	NO	Surgical removal of the tumor was followed by clinical recovery and normalization of inflammatory indices.
Ren et al. (10)	46	Male	CT revealed a well demarcated homogenous mass in the parenchyma of the right lower lobe of lung, suspicious of malignant tumor. PET/CT did not reveal tumor elsewhere.	NO	After resection of the right lower lobe of lung, the patient was well with no evidence of recurrence at 1 year.

This case involved a middle-aged female patient with a lesion growing in the right middle lobe bronchus, presenting with recurrent paroxysmal dry cough as the primary symptom. Bronchoscopy revealed nodular or polypoid new organisms that grew rapidly, leading to secondary obstructive pneumonia or atelectasis within 1 week. Higna M.R. et al. (16) reported that PAFH can grow in the pulmonary artery, located in the pulmonary trunk and above the lobe, with a clear boundary, and the mass may protrude locally outside the pulmonary artery, showing moderate or significant enhancement on CT. PAFH is often depicted on CT as low-density or mixed density mass shadows in the lung, with clear boundaries, cystic changes, and necrosis, rarely bleeding, and obvious enhancement of the solid part on the enhanced scan. 18F-FDG PET/CT imaging of pulmonary AFH has been reported in a small number of cases (17) (Table 1). PET/CT showed significant uptake of 18F-FDG by tumors. The SUVmax for this patient was 11.3, similar to reported cases (17, 18). PAFH exhibits a higher uptake of 18F-FDG, which is speculated to be related to its pathological components, which contain a large number of lymphocytes and have a higher uptake of FDG. PET/CT has some value in PAFH as it helps to stage the tumor and develop the best treatment plan. In this case, C-reactive protein was elevated, which was similarly reported by Higna M.R. et al. (16). Among them, two cases were found to have anemia and elevated inflammatory indicators by laboratory examination, which may be related to the production of cytokines such as IL-2 and IL-6 by tumor cells. None of the patients in this study had mediastinal lymph nodes or distant metastasis, and no signs of recurrence or metastasis were found during the 2-year follow-up, which is consistent with the characteristics of low-grade malignant tumors of PAFH. Based on the imaging and laboratory examination of this case, the PET/CT findings were bronchial occlusion in the middle lobe of the right lung, atelectasis, and a nodular soft tissue density shadow with uniform density, high uptake of 18F-FDG, and mediastinal lymph node enlargement. Considering the diagnostic ideas of common diseases and the lack of imaging experience in PAFH, it was misdiagnosed as lung cancer. The imaging findings of PAFH lack specificity, and it can also be distinguished from pneumonia myofibroblastoma, bronchiolung cancer, and pulmonary myxosarcoma. Pneumonia myofibroblastoma is mostly located in the superficial part of the lung with long burrs at the edge. CT accompanied by the “flatness sign” is helpful in distinguishing it. Histology showed that the alveolar nucleus and nucleolus of tumor cells were prominent, and they were mixed with lymphocytes and plasma cells, and did not form a lymphatic-like structure. Immunohistochemical markers SMA and ALK were partially positive, and ALK gene rearrangement was possible (19). There is overlap in the imaging of bronchiolung cancer and PAFH, but the tumor tissue of bronchiolung cancer is friable and necrotic, and it is prone to form cavities or cancerous lung abscesses. Its CT scan shows irregular or quasi-circular hilar masses. The lumps often show lobulated signs and marginal umbilical concave changes, which are easy to cause bronchial tube wall thickening, lumen stenosis, and bronchial truncation. The enhanced scan is mild to moderate and the enhancement is uneven. The clinical manifestations, histological morphology, immunophenotype, and molecular genetic changes of pulmonary myxoid sarcoma and PAFH overlap, and some scholars believe that the two may be the same lesion lineage (20). Invasive growth and necrosis are more common in pulmonary myxoid sarcomas. Extensive mesenchymal myxosis is characterized by a reticular or lace-like arrangement of tumor cells and increased atypical mitosis. The PAFH boundary is clear, the tumor cell morphology is mild, and the mitotic image is very rare. However, it is challenging to differentiate PAFH from lung cancer, sarcoma, or other tumors solely based on imaging findings, and the final diagnosis depends on histopathological examination. Currently, the primary treatment for PAFH is surgical resection, and some cases may require radiotherapy. Preoperative radiotherapy is generally preferred over postoperative radiotherapy. Due to the risk of local recurrence in 2–12% of cases (4, 8)(Table 1), close postoperative follow-up is crucial. In this patient, 2 years of postoperative follow-up revealed no abnormalities.

In summary, primary pulmonary angiomatoid fibrous histiocytoma (PAFH) is exceptionally rare, and its clinical and imaging presentations lack distinct characteristics, necessitating a definitive diagnosis through pathology. Nevertheless, when middle-aged patients present with endobronchial soft tissue shadows, clear boundaries, uneven density, and moderate to significantly enhanced soft tissue masses on contrast-enhanced scans, along with high fluorodeoxyglucose (FDG) uptake on PET/CT, PAFH should be included in the differential diagnosis. Surgical resection remains the optimal treatment for PAFH. PET/CT imaging holds value in the diagnosis of PAFH and is highly applicable for preoperative staging, postoperative efficacy assessment, and follow-up. In conclusion, this case report contributes to the expanding knowledge of lung and bronchial tumors.

## Data availability statement

The original contributions presented in the study are included in the article/supplementary material, further inquiries can be directed to the corresponding author.

## Ethics statement

Written informed consent was obtained from the individual(s) for the publication of any potentially identifiable images or data included in this article.

## Author contributions

MS: Data curation, Writing – original draft, Writing – review & editing. SF: Data curation, Writing – original draft. WQ: Data curation, Writing – review & editing. ZL: Data curation, Writing – original draft. RX: Data curation, Writing – review & editing. FL: Data curation, Writing – original draft.
